# Supplementary material for: Harnessing photoinduced electron transfer to optically determine protein sub-nanoscale atomic distances
Source: Nat Commun. 2018 Nov 9;9:4738. doi: 10.1038/s41467-018-07218-6 (PMC6226468; doi:10.1038/s41467-018-07218-6)
Supplement: Supplementary file 1 — Supplementary Information [file 41467_2018_7218_MOESM1_ESM.pdf]

Harnessing Photoinduced Electron Transfer to Optically Determine Protein Sub-nanoscale Atomic Distances

Pantazis *et al*.

SUPPLEMENTARY FIGURE 1

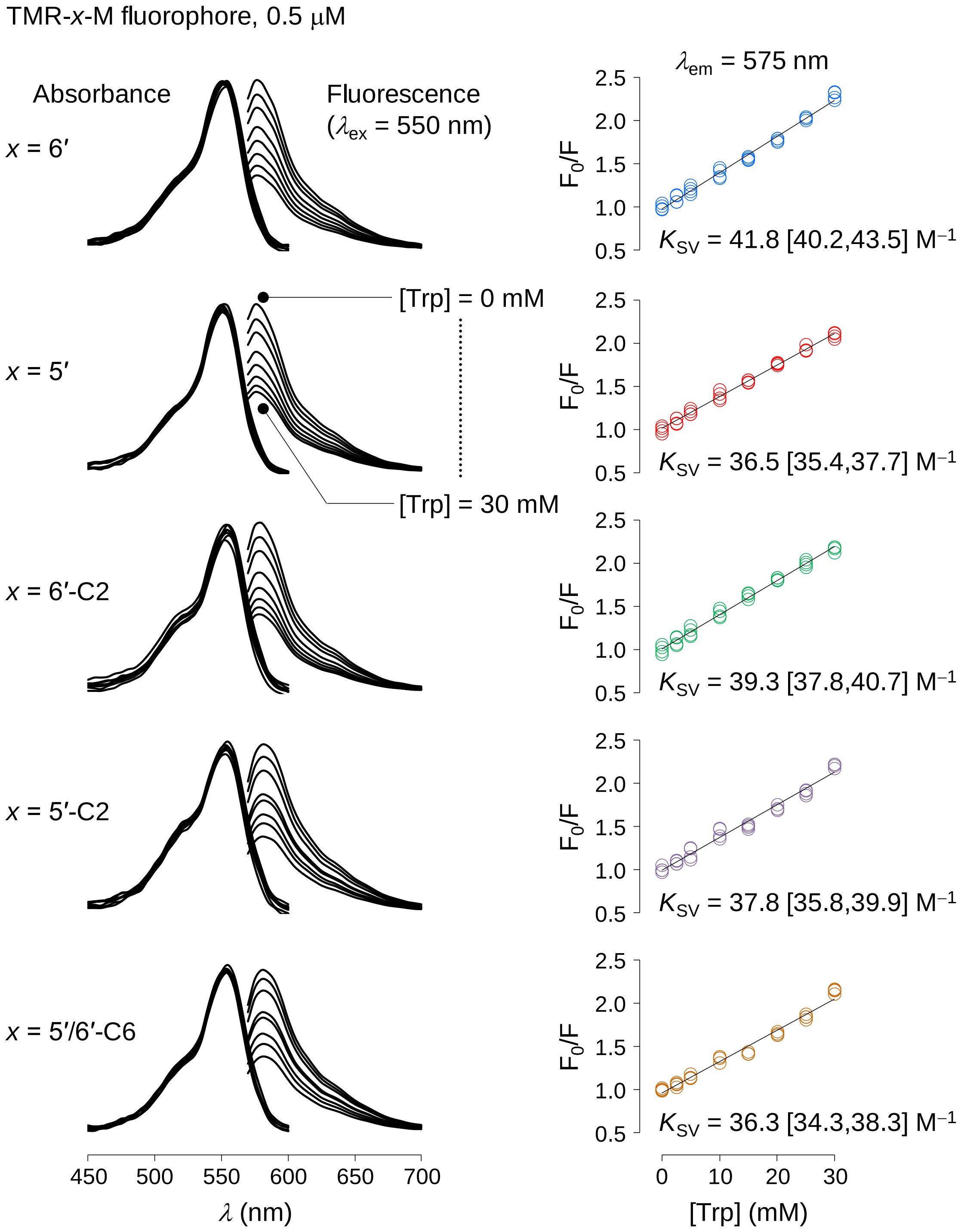

Supplementary Figure 1: Trp is an equally efficient quencher of TMRM fluorophores of different lengths. Absorbance and fluorescence spectra of TMRM fluorophores of different lengths, in increasing [Trp]. Stein-Volmer plots[1] are shown on the right, used to evaluate the TMRM-Trp bimolecular quenching constant, K_{SV}. n=4 measurements per fluorophore and [Trp]. R^2 for all fittings was 0.98-0.99.

SUPPLEMENTARY FIGURE 2

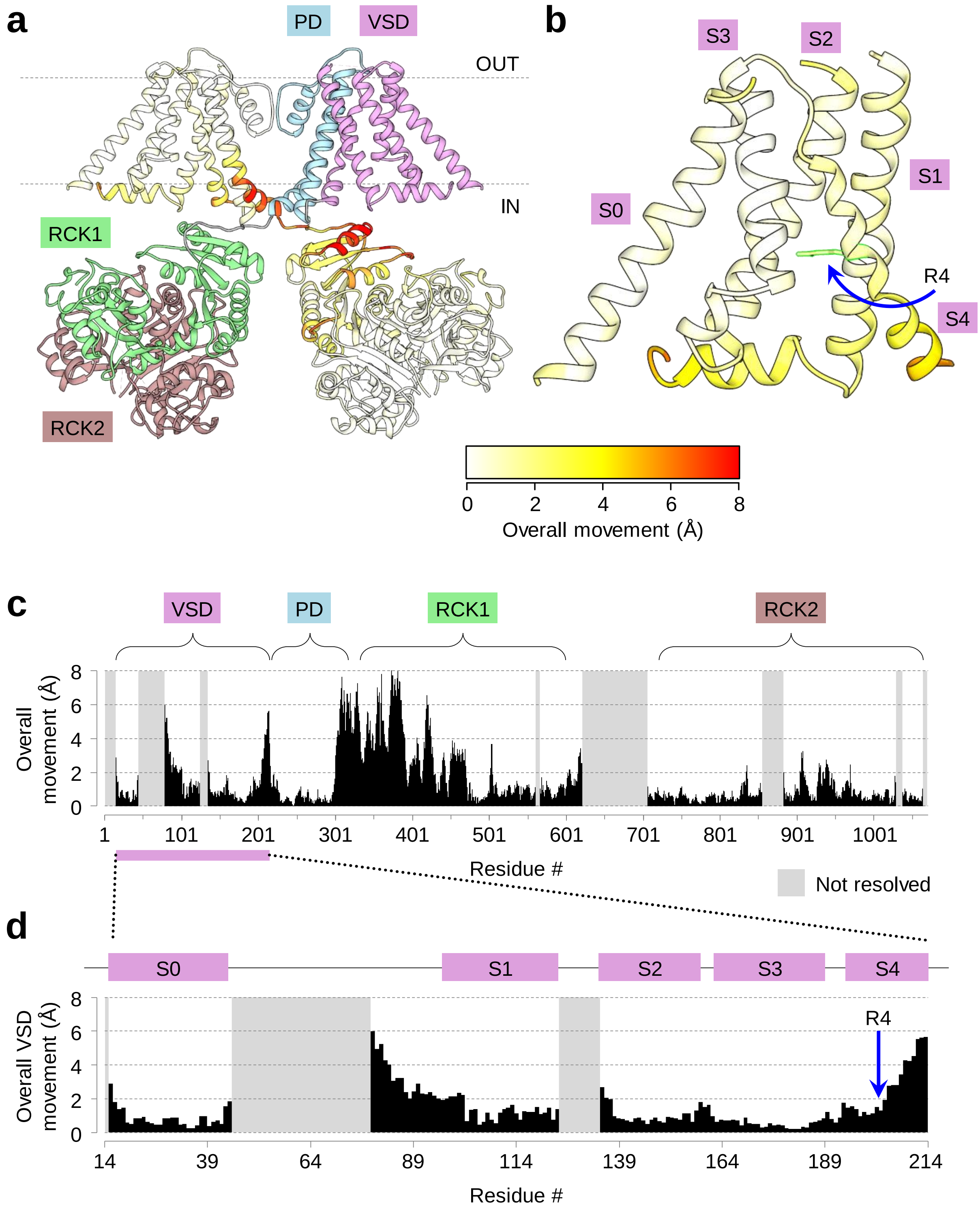

Supplementary Figure 2: BK channel modular structure and ligand-evoked conformational changes. **(a)** BK channel structure (two out of four α subunits shown for clarity) (PDB: #5TJI [2]). One subunit is colored according to constituent functional domain (voltage-sensing domain, VSD:

pink; pore domain, PD: light blue; cytosolic regulator of conductance for potassium 1, RCK1: light green; RCK2: brown). The other subunit is color-coded for intra-domain movement as a result of Ca^{2+}- and Mg^{2+}-binding, and channel opening. An approximate position of the lipid bilayer is shown in dashed lines. **(b)** An isolated BK VSD is shown in close-up, color-coded for relative intra-domain movement in closed and open channels. The voltage-sensing arginine on helix S4 (equivalent to R4 in Shaker K^+ channels[3]) is labeled and shown with its side-chain. **(c)** Pairwise intra-domain C_α-C_α movement between ligand-free / closed (PDB: #5TJI [2]) and Ca^{2+}- and Mg^{2+}-bound / open (PDB: #5TJ6 [4]) BK channels of *Aplysia californica*. **(d)** A more detailed look at the voltage-sensing region, also showing the spans of VSD transmembrane helices S0-S4. The position of voltage-sensing Arg in S4[3,5] is indicated as R4. Contrary to regions pertinent to Ca^{2+}/Mg^{2+} binding and pore opening, the voltage-sensing domain is practically in the same conformation in the two BK structures. This is not surprising given that strong membrane depolarization is required to activate the VSDs of these channels[6].

SUPPLEMENTARY FIGURE 3

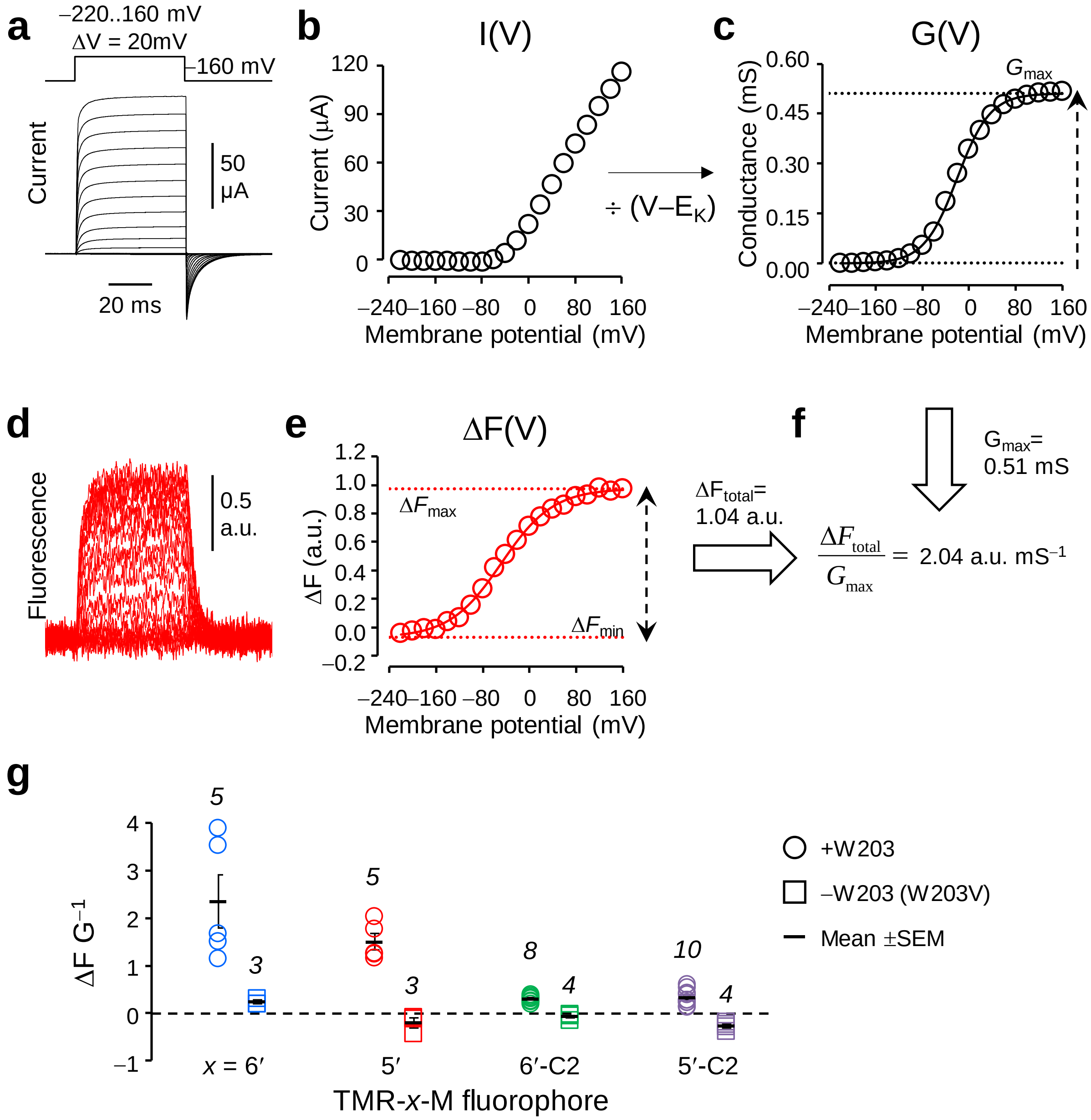

Supplementary Figure 3: Initial analysis of DEPET data: determination of $\Delta F\ G^{-1}$ values. **(a)** A family of 50-ms voltage steps in the cut-open oocyte voltage clamp[7,8] elicits K^+ current from human BK channels expressed in the membrane of *Xenopus* oocytes. **(b)** The macroscopic current at the end of the pulse is measured and plotted against the membrane potential (I(V) plot). **(c)** The current is divided by the driving force ($E_K \approx -65$ mV) to produce macroscopic conductance (*G*); the latter is fit by the sum of two Bolzmann distributions to provide the limiting macroscopic conductance parameter G_{max}, utilized as a measure of channel expression. **(d)** Ensemble fluorescence is recorded at the same time as the membrane current from the same area of the oocyte membrane. In this experiment, the fluorescence comes from TMR-5′-M labels conjugated at position 136, extracellular to helix S1. Note that membrane depolarization elicits not only BK

channel pore opening (K^+ current), but also fluorescence deflections (ΔF), which reflect conformational rearrangements taking place in the vicinity of S1, in the channel voltage-sensing domains. These deflections are practically abolished by mutation W203V (Figure. 2b & Supplementary Figure 3g), indicating that they arise from the state-dependent interaction of the fluorophore with the quenching W203, at the extracellular flank of S4 (Fig.2c). **(e)** The ΔF signal is fit to a Boltzmann distribution. The limiting minimal and maximal ΔF are used to calculate the total fluorescence change, ΔF_{total}. **(f)** Combining the G_{max} and ΔF_{total} measurements produces a measure of fluorescence change (differential state-dependent Trp-induced quenching) normalized for channel expression. $\Delta F\ G^{-1}$ observations are resampled with replacement (bootstrapping[9]) and simultaneously fit together with measurements of other TMRM labels to their respective FDQ functions and extract distance information. **(g)** Collected $\Delta F\ G^{-1}$ measurements from BK channels labeled at position 136 (extracellular to helix S1), with (circles) or without (squares) residue W203. Each point represents the $\Delta F\ G^{-1}$ data from an oocyte, extracted from simultaneously-recorded current and fluorescence deflections. The number of observations (oocytes) is reported above each dataset. Means are shown as dashes, and error bars represent ±1 SEM. Representative traces are shown in panel d and Fig.2a-b.

SUPPLEMENTARY FIGURE 4

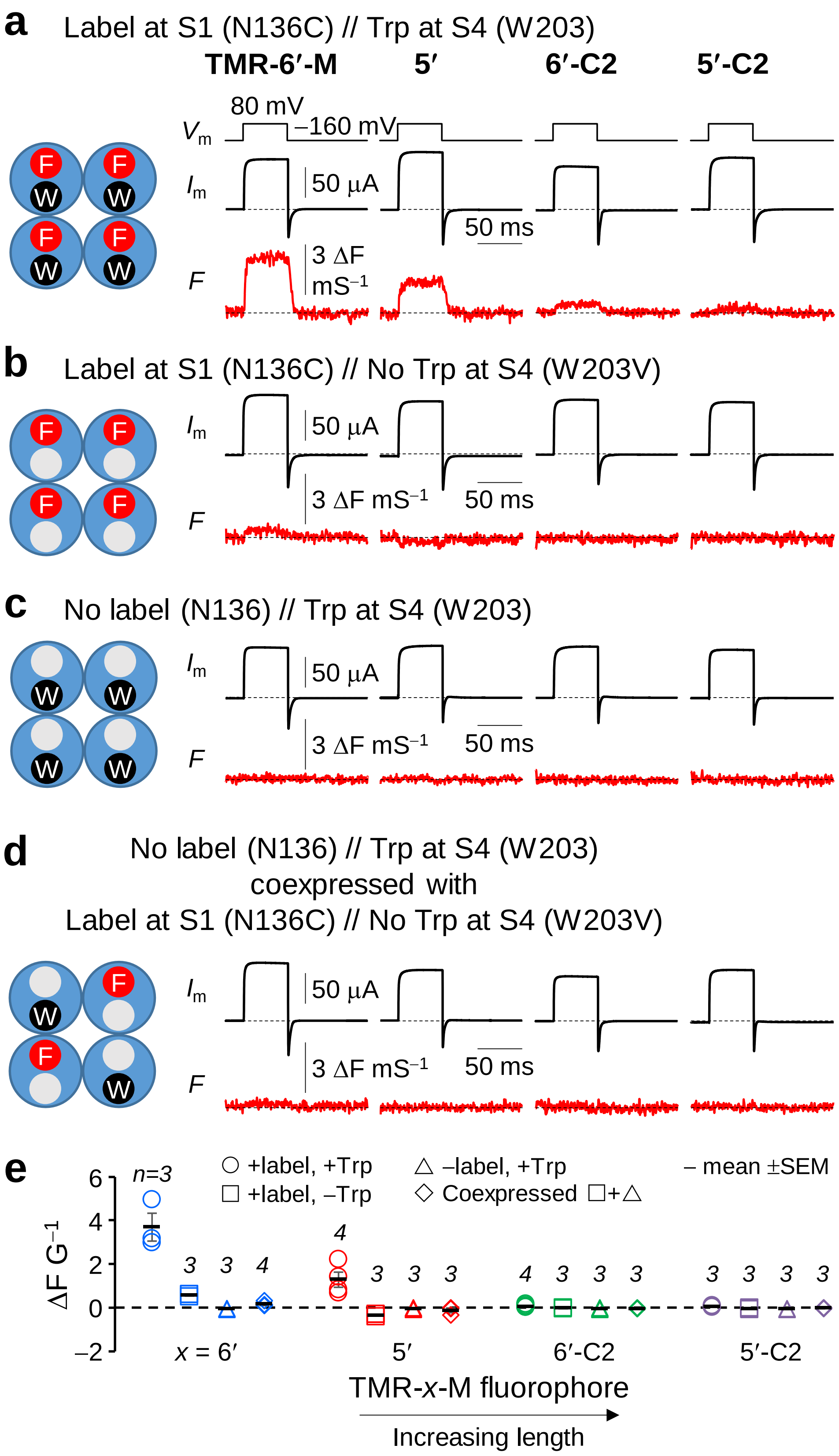

Supplementary Figure 4: Inter-subunit quenching does not contribute to the observed voltage-evoked ΔF. **(a,b)** As in Fig.2a,b: depolarization-evoked BK channel currents (black) and fluorescence traces (red) from channels labeled at position 136 (helix S1) with fluorophores of increasing length. **(c)** The same fluorometry experiment in oocytes expressing BK subunits without an extracellular cysteine results in no voltage-dependent ΔF. **(d)** Coexpressing subunits with (i) no extracellular Cys and an intact S4 Trp (W203) and (ii) an extracellular Cys (N136C) but no S4 Trp (W203V) also results in minimal fluorescence change. This experiments shows that the ΔF signals reported from subunits with both Cys and Trp are not contaminated by inter-subunit quenching, from either an adjacent subunit in the same channel, or from another nearby channel. **(e)** Experiments summary. All experiments in this figure were conducted in the same batch of oocytes, which was different from that of experiments shown in Figure 2a,b and Supplementary Figure 3g. Number of observations is reported above the data (open symbols) and their mean (dashes). Note that the proportion of ΔF by TMRM fluorophores of different length when labeling a BK channel subunit with Cys and Trp (circles) is very similar to that of a previous experiment (Supplementary Figure 3g). Errors bars represent ±1 SEM.

SUPPLEMENTARY FIGURE 5

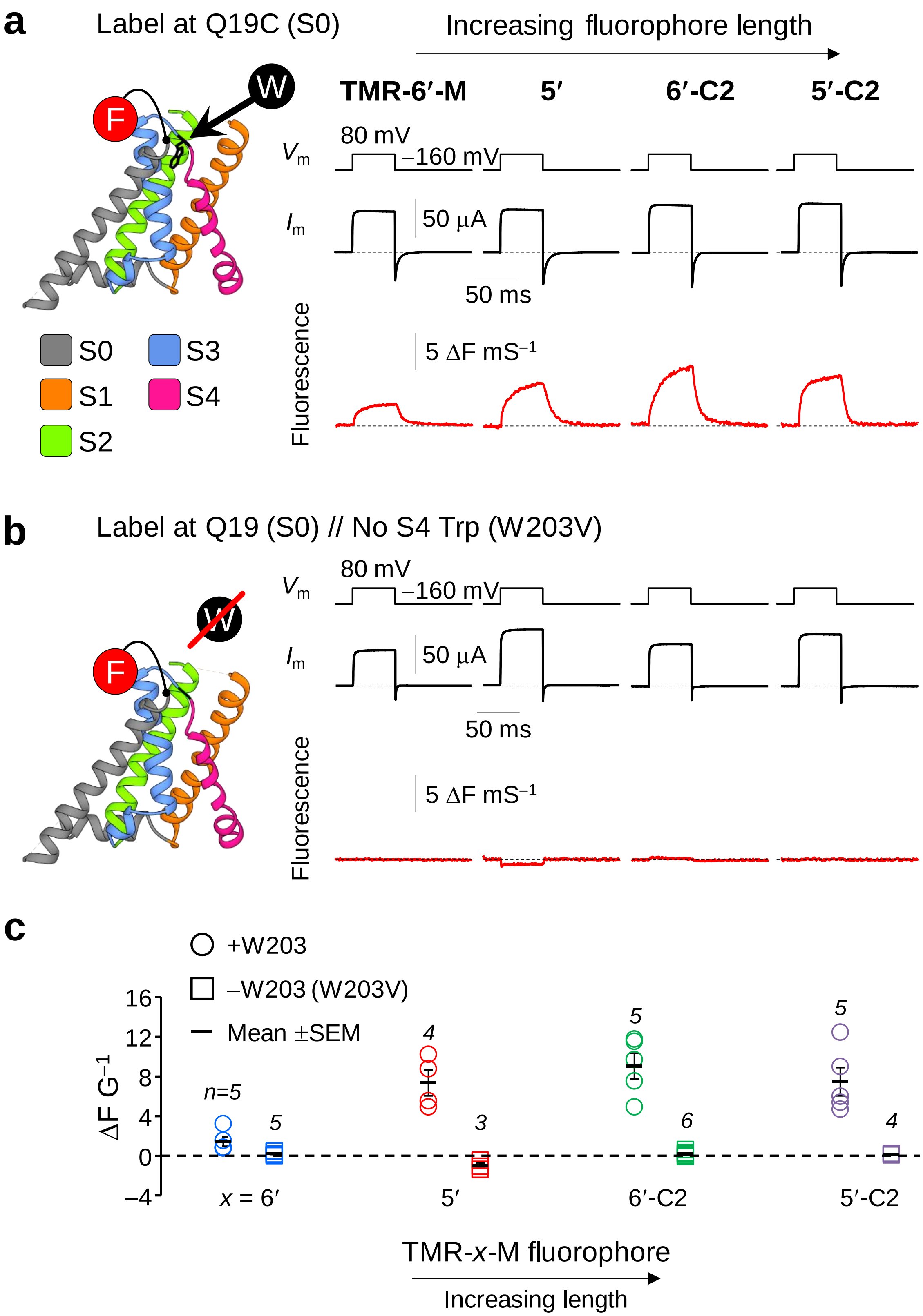

Supplementary Figure 5: W203, at the extracellular flank of BK transmembrane helix S4, differentially quenches TMRM fluorophores of different length conjugated at helix S0. **(a)** Simultaneously-acquired K^+ current (black) and fluorescence (red) from oocytes expressing human BK channels fluorescently labelled outside helix S0 (position 19) with different TMRM fluorophores, upon a 50-ms voltage pulse from –160 to +80 mV. Note that the pattern of voltage-dependent fluorescence change (ΔF) is different from that of labels at S1 (Fig.2a), indicating that the S0-S4 distance is different from S1-S4. **(b)** As above, when the native Trp extracellular to S4 in removed (W203V), ΔF is strongly diminished. **(c)** Total voltage-dependent ΔF normalized by maximal macroscopic conductance for each TMRM fluorophore labeling position 19, in the presence (circles) or absence (squares) of W203. The number of observations is reported above the symbols. Means are shown as dashes, and error bars represent ±1 SEM. The DEPET fit of these data, and derived distances are reported in Fig.4a-c and Supplementary Table 3. The orientation of W203 side-chain with respect to position 145 is mapped in Fig.5a.

SUPPLEMENTARY FIGURE 6

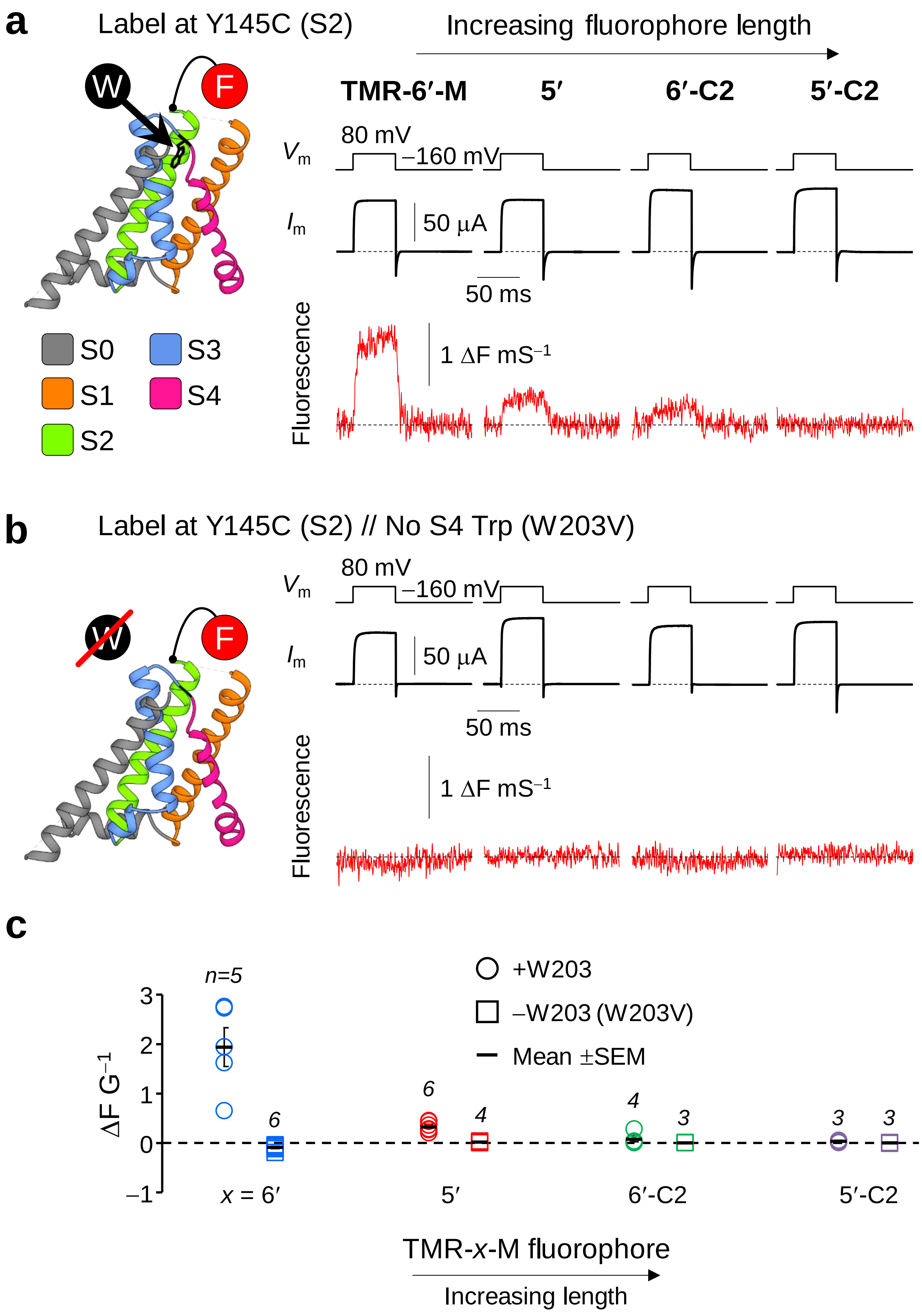

Supplementary Figure 6: W203, at the extracellular flank of BK transmembrane helix S4, differentially quenches TMRM fluorophores of different length conjugated at helix S2. **(a)** Simultaneously-acquired K^+ current (black) and fluorescence (red) from oocytes expressing human BK channels fluorescently labelled outside helix S2 (position 145) with different TMRM fluorophores, upon a 50-ms voltage pulse from –160 to +80 mV. **(b)** As above, when the native Trp extracellular to S4 in removed (W203V), ΔF is strongly diminished. **(c)** Total voltage-dependent ΔF normalized by maximal macroscopic conductance for each TMRM fluorophore labeling position 145, in the presence (circles) or absence (squares) of W203. The number of observations is reported above the symbols. Means are shown as dashes, and error bars represent ±1 SEM. The DEPET fit of these data, and derived distances are reported in Fig.4g-i and Supplementary Table 3. The orientation of W203 side-chain with respect to position 145 is mapped in Fig.5a.

SUPPLEMENTARY FIGURE 7

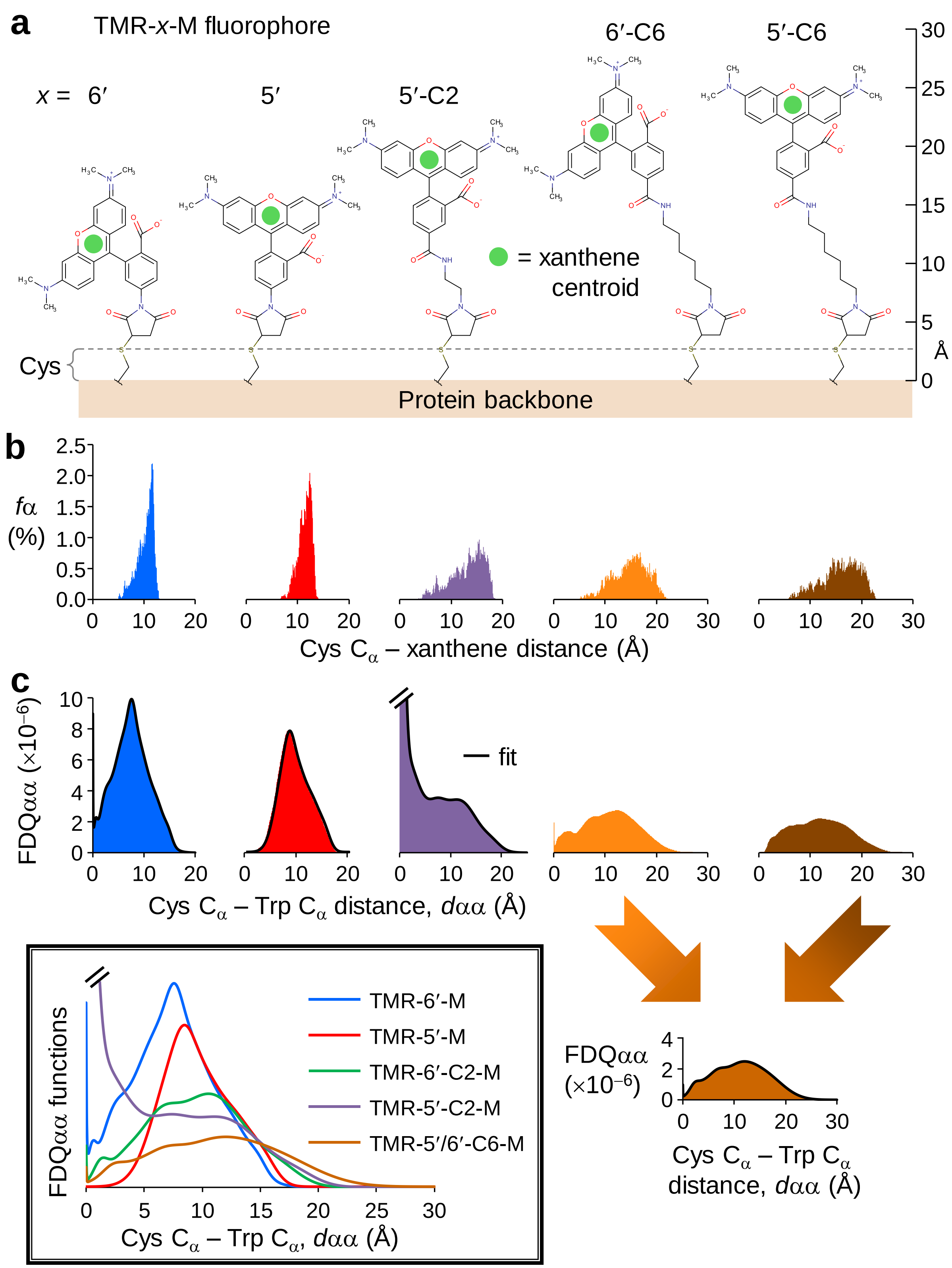

Supplementary Figure 7: Calculating FDQ functions for TMRM labels of different lengths. **(a)** Cys-TMRM conjugates of different length, shown aligned with respect to the Cys C_α atom, part of the protein backbone. The xanthene moiety (centroid in green filled circle) stretches different lengths from the Cys C_α, according to fluorophore orientation and linker length. **(b)** As in Fig.3b, molecular dynamics simulation of the Cys-TMRM conjugates yield the probability to encounter (and therefore quench) the xanthene at a given distance from the Cys C_α (f_α function). **(c)** As in Fig.3c, the f_α functions of each fluorophore are combined with the chance to encounter the Trp indole (q_α function) over a given distance separating the Cys and Trp C_α atoms: the $FDQ_{\alpha\alpha}$ function. Note that the TMR-5′-C6-M and TMR-6′-C6-M fluorophores were only available as mixed isomers, so their FDQ functions are also mixed. The inset shows the superimposed $FDQ_{\alpha\alpha}$ functions of all fluorophores. The parameters of the $FDQ_{\alpha\alpha}$ functions are reported in Supplementary Table 1. A rendering of the $FDQ_{\alpha\alpha}$ function with respect to the BK channel structure is shown in Supplementary Figure 8.

SUPPLEMENTARY FIGURE 8

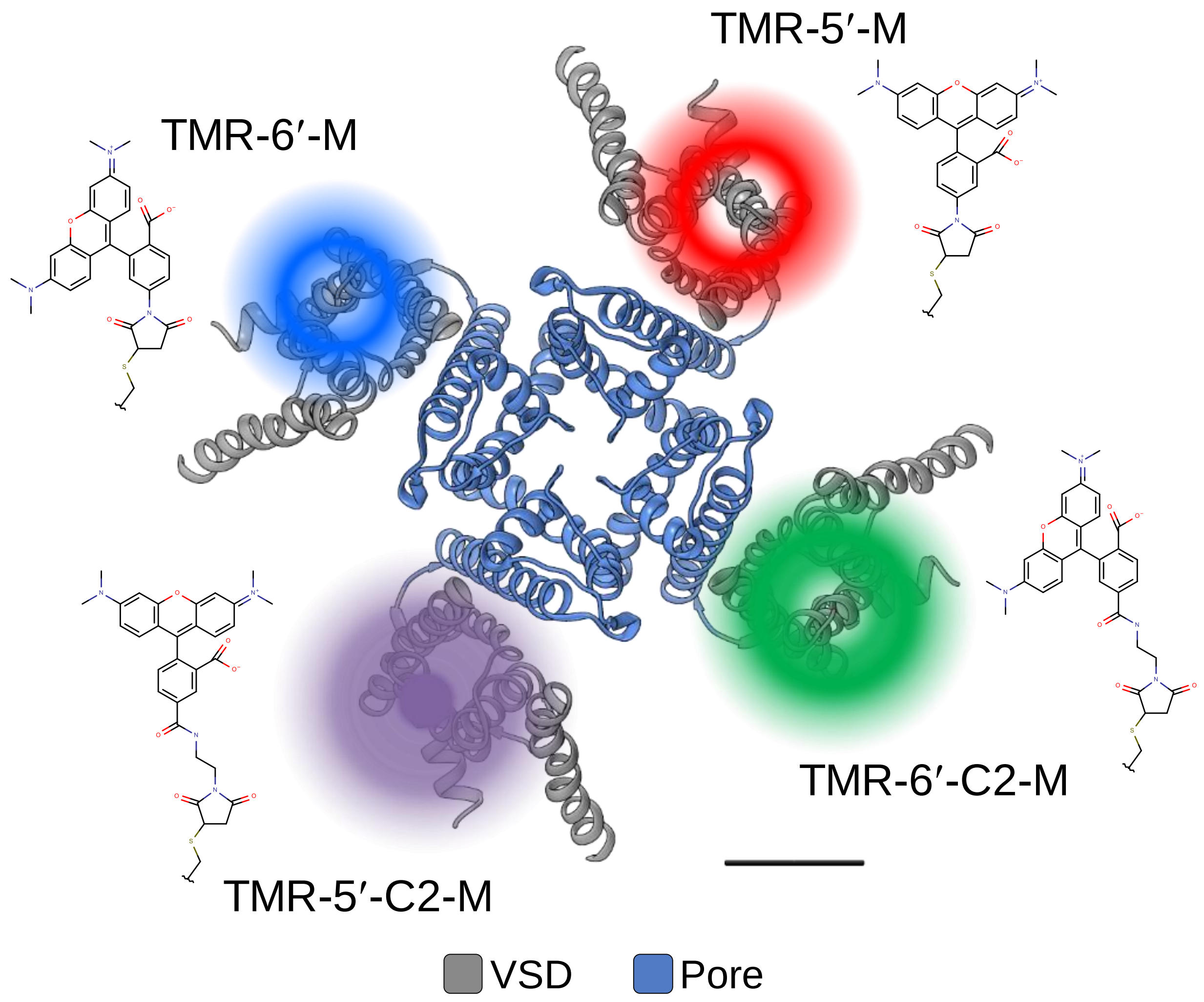

Supplementary Figure 8: TMRM fluorophore quenching distance functions ($FDQ_{\alpha\alpha}$) compared to the BK channel structure. A top view of a homotetrameric BK channel (orthographic projection) is shown (PDB: #5TJI [2]); the cytosolic domains have been omitted for clarity. Each voltage-sensing domain (VSD) is in gray, while the central K^+-selective pore is shown in blue. Each VSD has been "labeled" with a TMRM fluorophore of different length: these are the same probes used in the fluorometry experiments (Figure 2 and Supplementary Figures 4, 5, 6). Note that, in the fluorometry experiments, all subunits in the oocyte membrane are labeled with the same TMRM fluorophore. The distance-dependent quenching probability of each probe by Trp ($FDQ_{\alpha\alpha}$ function; Figure 3c and Supplementary Figure 7c) is rendered on each VSD, centered on the C_α atom of position 123 on helix S1 (homologous to position 136 of the human BK channel): increasing color density represents higher quenching probability. The scale bar is 20 Å and applies to both the structure and the $FDQ_{\alpha\alpha}$ functions.

SUPPLEMENTARY FIGURE 9

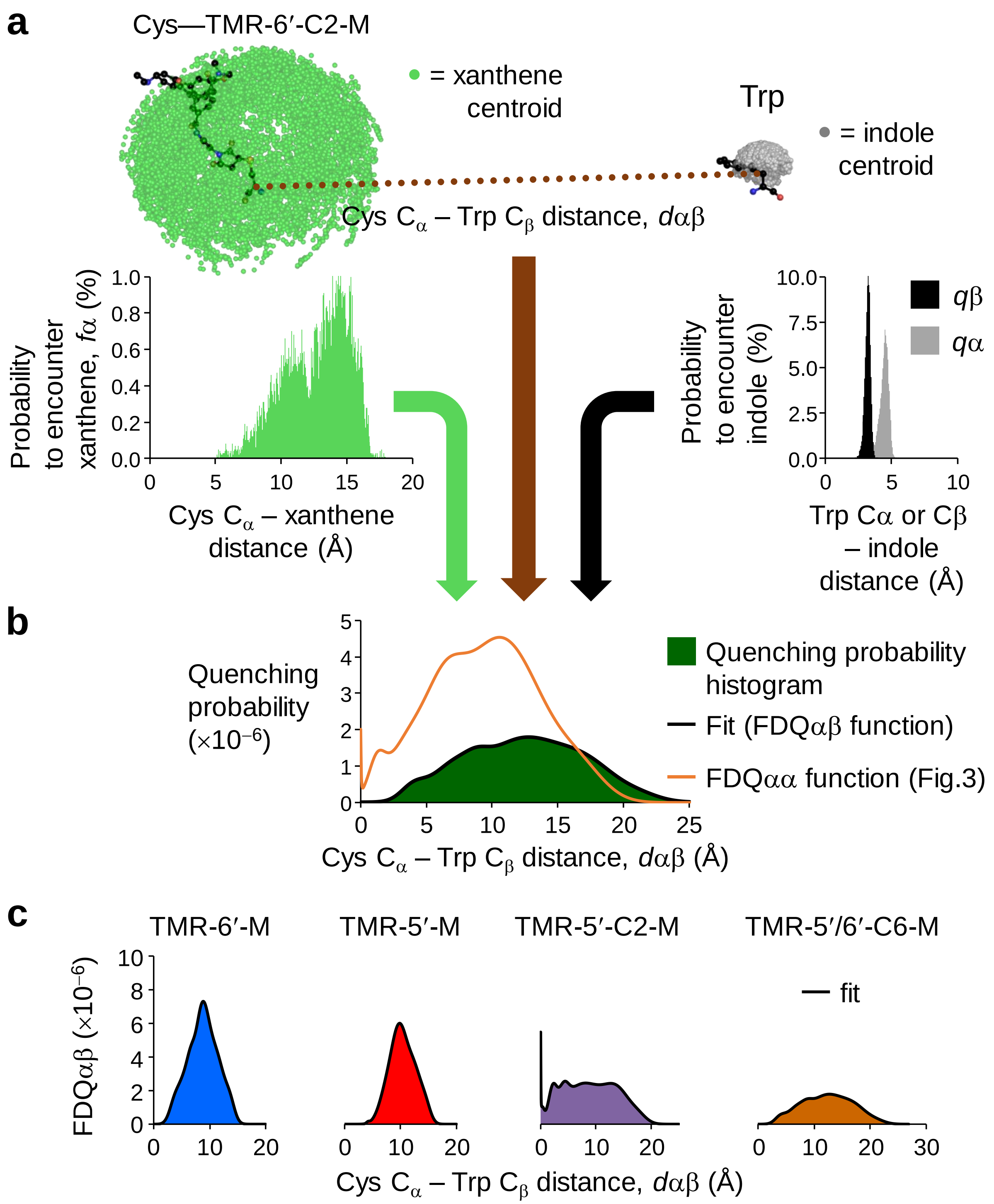

Supplementary Figure 9: Calculating PET quenching probability as a function of Cys C_α / Trp C_β distance. **(a)** As in Fig.3b, molecular dynamics simulations of Cys-TMR-6′-C2-M are used to quantify the chance to encounter xanthene a given distance from the Cys C_α (f_α, green). In the case

of Trp simulations, the distance separating indole from the C_β atom (the first atom of the Trp side-chain) can be binned into distribution q_β (black). The indole-C_α distance distribution (q_α; Fig.3b) is also shown for comparison, in patterned grey. **(b)** As in the $FDQ_{\alpha\alpha}$ calculation (Fig.3c), f_α is intersected with q_β, to yield distribution $FDQ_{\alpha\beta}$ (dark green), or the PET quenching probability over separating distance $d_{\alpha\beta}$, between the Cys C_α and the Trp C_β atoms. The $FDQ_{\alpha\alpha}$ function for the TMR-6′-C2-M label is also plotted for comparison (orange; Fig.3c). **(c)** $FDQ_{\alpha\beta}$ distributions for the other TMRM fluorophores. Fitting parameters are reported in Supplementary Table 2.

SUPPLEMENTARY FIGURE 10

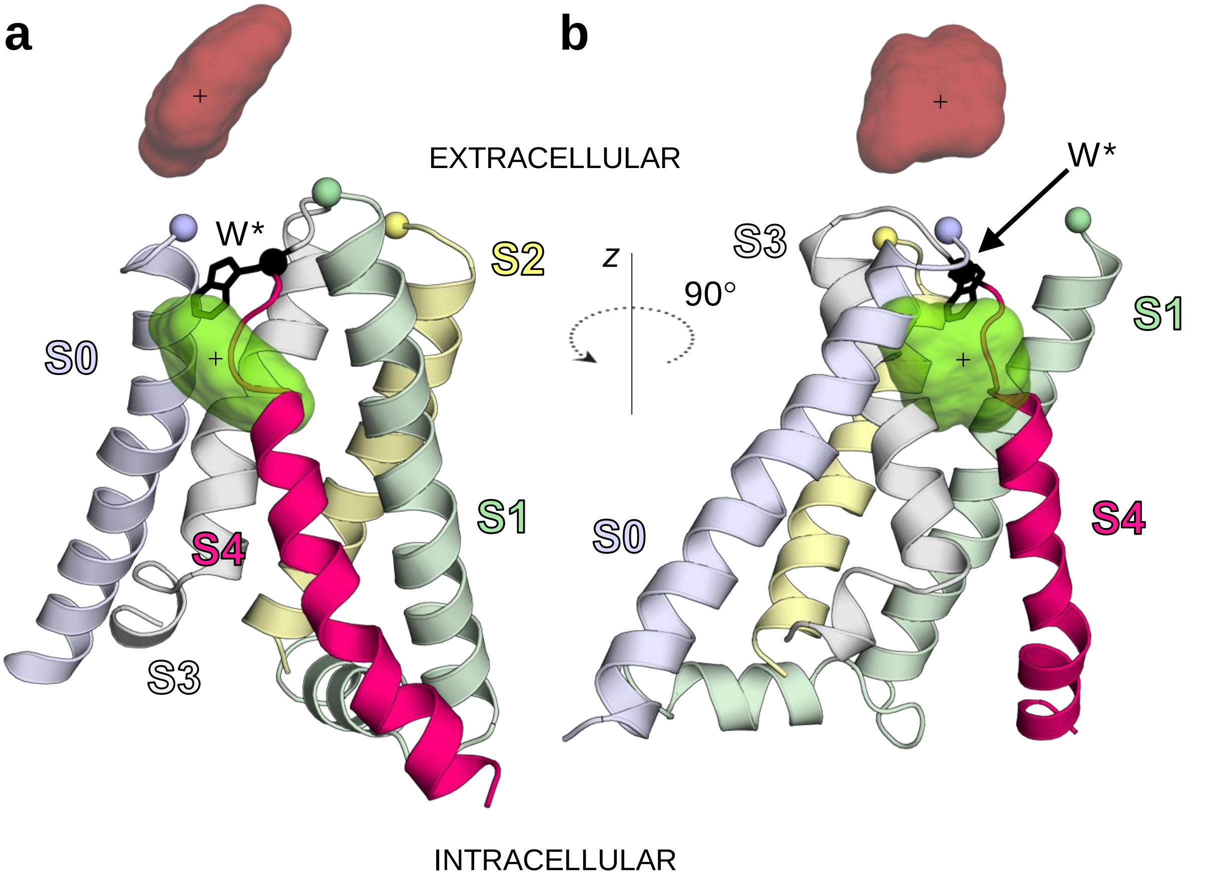

Supplementary Figure 10: Combination of DEPET and cryo-EM data to model the BK VSD activation transition. **(a)** The cryo-EM-resolved structure of the *Aplysia* BK VSD (ligand-free channel; PDB: #5TJI [2]) is shown. The homologous Trp used as fluorescence quencher in this study is shown in black (W192, *W**), its C_α atom indicated by the black sphere. The nearest or most homologous positions resolved in the structure to those fluorescently labeled for DEPET experiments are shown as blue (helix S0), green (S1) and yellow (S2) spheres. Assuming this to be a structure of the VSD in the Resting state, trilateration was performed to determine the position of W* in the Active state, by imposing DEPET constraints: specifically, the distance change of the Trp C_α from positions surrounding it upon activation ($d_{\alpha\alpha,A}$–$d_{\alpha\alpha,R}$, or δ distributions; mean & 95% C.I. reported in Supplementary Table 3a). Two sets of solutions result from this process, reflecting a ~12 Å outward S4 movement, or a ~7 Å inward movement, shown as red and green volumes, respectively. The geometric means of these distributions are indicated by black crosses. The inward S4 movement solution (green volume) may be rejected, since the positively-charged S4 segment is expected to move outwards upon membrane depolarization. **(b)** The same elements as in **a**, rotated by 90° counterclockwise around the *z* axis, normal to the membrane.

Fluorophore	TMR-6′-M	TMR-5′-M	TMR-6′-C2-M	TMR-5′-C2-M	TMR-5′/6′-C6-M
α_1	7.8885E-06	0	1.7384E-06	2.8128E-04	7.6610E-07
δ_1	4.3048E-02	n/a	3.7683E-02	2.8241E-02	3.1186E-02
α_2	0	0	0	1.4295E-04	0
δ_2	n/a	n/a	n/a	0.12964	n/a
A_1	8.8045E-07	1.4367E-06	5.6214E-06	3.0031E-05	2.1342E-06
μ_1	0.52098	11.793	6.4125	11.607	6.7523
σ_1	0.33861	1.4332	1.3946	3.5642	1.4206
A_2	3.6013E-06	3.8160E-06	6.2109E-06	5.7974E-05	4.4682E-06
μ_2	12.986	13.588	3.6506	9.1061E-02	3.6570
σ_2	1.2969	1.1325	1.7278	3.4520	2.0466
A_3	2.8402E-06	2.3413E-05	1.1746E-06	2.7769E-05	6.3457E-07
μ_3	2.2905	10.372	1.2214	7.1077	2.2212
σ_3	0.88304	2.1160	0.60280	1.6887	0.80104
A_4	7.0164E-05	1.9057E-05	3.5925E-05	1.5142E-06	1.6606E-05
μ_4	7.3799	7.2328	10.628	18.165	10.551
σ_4	3.3305	1.7845	3.1778	1.6584	3.4328
A_5	9.8466E-07	2.8998E-06	2.8023E-06	0	1.2156E-05
μ_5	14.843	15.387	16.774	n/a	16.180
σ_5	0.73683	1.0527	1.7742	n/a	3.5925
A_6	2.5625E-06	3.6468E-06	0	0	0
μ_6	7.6077	8.4728	n/a	n/a	n/a
σ_6	0.67034	0.94975	n/a	n/a	n/a

Supplementary Table 1: Parameters for the empirical fitting of the PET quenching probability of each fluorophore as a function of distance between the labeled Cys and the Trp C_α atoms ($FDQ_{\alpha\alpha}$ functions; eq. 14; Fig. 3 & Supplementary Figure 7). The function is the sum of up to two exponential decay components (α: amplitude; δ: length constant) and six Gaussian distribution components (A: amplitude; μ: mean; σ: standard deviation). The δ, μ and σ parameters are in units of Å.

Fluorophore	TMR-6′-M	TMR-5′-M	TMR-6′-C2-M	TMR-5′-C2-M	TMR-5′/6′-C6-M
α_1	0	0	0	4.6757E-06	0
δ_1	n/a	n/a	n/a	0.040064	n/a
α_2	0	0	0	0	0
δ_2	n/a	n/a	n/a	n/a	n/a
A_1	4.4886E-06	5.4594E-06	2.0047E-05	1.1912E-06	4.8594E-06
μ_1	5.5249	7.3925	11.919	17.749	17.313
σ_1	0.88037	1.3118	2.4317	1.4172	2.2244
A_2	1.9919E-06	9.0931E-06	3.6396E-07	2.1570E-06	5.7526E-07
μ_2	13.672	12.552	2.7892	4.1502	21.274
σ_2	0.82933	1.2760	0.49171	0.85671	1.5329
A_3	3.5288E-06	1.8931E-05	1.0230E-06	6.0932E-07	3.6566E-06
μ_3	6.6376	9.8032	4.2346	0.24714	6.9878
σ_3	0.72781	1.3918	0.83449	0.34427	1.8012
A_4	3.7762E-06	1.7900E-06	4.6022E-07	2.7729E-06	1.2608E-05
μ_4	3.9235	14.536	5.6402	2.1766	12.280
σ_4	0.96509	0.88166	0.67871	0.70707	2.9649
A_5	1.3628E-05	5.7016E-08	1.7678E-06	1.0200E-05	7.8243E-07
μ_5	11.249	4.1746	16.388	13.895	3.9353
σ_5	1.4273	0.27126	1.3388	2.3244	0.92259
A_6	1.9908E-05	0	8.4773E-06	2.1528E-05	3.5934E-07
μ_6	8.6160	n/a	7.5867	7.6596	8.8675
σ_6	1.2243	n/a	1.6802	3.5962	0.82826

Supplementary Table 2: Parameters for the empirical fitting of the PET quenching probability of each fluorophore as a function of distance between the labeled Cys C_α and the Trp C_β atoms ($FDQ_{\alpha\beta}$ functions; Supplementary Figure 9). The function is the sum of up to two exponential decay components (α: amplitude; δ: length constant) and six Gaussian distribution components (A: amplitude; μ: mean; σ: standard deviation). The δ, μ and σ parameters are in units of Å.

SUPPLEMENTARY TABLE 3

a

DEPET measurements (*H. sapiens*) — Cryo-EM structures (*A. californica*)

		Distance from W203 Cα (helix S4)				Distance from W192 Cα (helix S4)		
Helix	Position	Resting state, d_R	Active state, d_A	Distance change, δ	Position	Closed, ligand-free	Open, Ca^{2+}/Mg^{2+}	Distance change
S0	19	16.7 [16.1,17.3]	18.6 [17.3,21.5]	1.9 [0.2,5.1]	13 *(match)*	*not resolved*	*not resolved*	*not resolved*
					14 *(+1 res.)*	*not resolved*	12.5	*not resolved*
					15 *(+2 res.)*	7.0	8.7	1.7
S1	136	8.0 [7.3,8.8]	14.2 [13.5,15.1]	6.3 [5.4,7.2]	123 *(match)*	12.6	13.0	0.4
S2	145	6.6 [6.3,6.9]	13.0 [12.6,13.3]	6.4 [5.6,7.1]	134 *(match)*	*not resolved*	12.6	*not resolved*
					135 *(+1 res.)*	11.1	13.8	2.7

b

		Distance from W203 Cβ (helix S4)	
Helix	Position	Resting state, d_R	Active state, d_A
S0	19	15.6 [15.0,16.1]	17.2 [16.1,19.1]
S1	136	9.1 [8.5,10.0]	15.7 [14.9,16.6]
S2	145	8.1 [7.9,8.3]	14.0 [13.4,14.4]

	Distance from W192 Cβ (helix S4)	
Position	Closed, ligand-free	Open, Ca^{2+}/Mg^{2+}
13 *(match)*	*not resolved*	*not resolved*
14 *(+1 res.)*	*not resolved*	11.1
15 *(+2 res.)*	6.0	7.4
123 *(match)*	14.1	14.4
134 *(match)*	*not resolved*	13.2
135 *(+1 res.)*	11.7	14.6

Supplementary Table 3: DEPET-resolved distances and comparison with cryo-EM data. All distances in Å. **(a, blue)** DEPET C_α—C_α distances for positions at the extracellular flanks of S0, S1 and S2 from W203 at S4 (Fig.4b,e,h), in the human BK channel. Errors are 95% C.I. **(a, yellow)** Cryo-EM-resoved distances for positions at the extracellular flanks of S0, S1 and S2 from W192 at S4 for the *Aplysia* BK channel resolved in closed, ligand-free conformation (PDB: 5TJI)[2] and open, Ca^{2+} and Mg^{2+}-bound conformation (PDB: 5TJ6)[4]. Note that W203 (*H.s.*) is homologous to W192 (*A.c.*) Residues are numbered according to the *Aplysia* protein, followed by their homology to their human counterpart, according to the alignment at [4]: *Match:* homologous residue; +1 res. / +2 res.: 1- or 2-residues C-terminal to the human position, respectively. Distance changes (δ) were applied to the cryo-EM coordinates to determine the position of S4 in the active state, in Supplementary Figure 10. **(b)** as in sub-table **a**, above, reporting DEPET and cryo-EM-resolved distances between the Trp C_β atom and the C_α atom of residues in surrounding helices S0, S1 and S2 (Fig.4c,f,i). Cryo-EM-determined distances are shown for comparison, in the yellow field.

**Number of proline residues**	**1**	**2**	**3**	**4**	**7**
Nominal Cys C_α – Trp C_α distance (Å)	6.6	9.2	12.4	15.5	24.6
DEPET mean estimate (Å) *(n)*	5.1 *(2)*	9.7 ± 0.9 *(5)*	13.3 ± 0.3 *(4)*	14.4 ± 0.2 *(5)*	24.1 ± 1.0 *(4)*
DEPET mean |error| (Å)	1.5	1.8 ± 0.3	0.8 ± 0.3	1.1 ± 0.2	1.2 ± 0.8
Overall DEPET |error| (Å) min / max / **mean**	0.02 / 3.3 / **1.3 ± 0.2**				

Supplementary Table 4: Nominal (expected) and experimentally-determined lengths of polyproline peptides (Fig.6). On average, DEPET estimates were off by ~1.3 Å. Errors are ±1 SEM.

SUPPLEMENTARY REFERENCES

1 Lakowicz, J. R. *Principles of Fluorescence Spectroscopy*. 3rd edn, 954 (Springer, 2006).

2 Hite, R. K., Tao, X. & MacKinnon, R. Structural basis for gating the high-conductance Ca^{2+}-activated K^+ channel. *Nature* **541**, 52-57, (2017).

3 Ma, Z., Lou, X. J. & Horrigan, F. T. Role of charged residues in the S1-S4 voltage sensor of BK channels. *J Gen Physiol* **127**, 309-328, (2006).

4 Tao, X., Hite, R. K. & MacKinnon, R. Cryo-EM structure of the open high-conductance Ca^{2+}-activated K^+ channel. *Nature* **541**, 46-51, (2017).

5 Pantazis, A., Gudzenko, V., Savalli, N., Sigg, D. & Olcese, R. Operation of the voltage sensor of a human voltage- and Ca^{2+}-activated K^+ channel. *Proc Natl Acad Sci U S A* **107**, 4459-4464, (2010).

6 Stefani, E. *et al.* Voltage-controlled gating in a large conductance Ca^{2+}-sensitive K^+ channel (hslo). *Proc Natl Acad Sci U S A* **94**, 5427-5431 (1997).

7 Stefani, E. & Bezanilla, F. Cut-open oocyte voltage-clamp technique. *Methods Enzymol* **293**, 300-318 (1998).

8 Pantazis, A. & Olcese, R. in *Encyclopedia of Biophysics* (ed G.C.K. Roberts) 406-413 (Springer, 2013).

9 Calmettes, G., Drummond, G. B. & Vowler, S. L. Making do with what we have: use your bootstraps. *J Physiol* **590**, 3403-3406, (2012).
